# A Rare Phenomenon of Stercoral Ileal Perforation in a Pregnant Woman

**DOI:** 10.7759/cureus.41529

**Published:** 2023-07-07

**Authors:** Snehasis Das, Sagar Prakash, Julia Sunil, Oseen Shaikh, Gopal Balasubramanian

**Affiliations:** 1 Surgery, Jawaharlal Institute of Postgraduate Medical Education and Research, Puducherry, IND

**Keywords:** transmural necrosis, peritonitis, impacted fecolith, ileal perforation, stercoral ulcer

## Abstract

Perforation peritonitis is one of the most common emergency presentations in Indian hospitals. Stercoral perforations are rare due to increased intraluminal pressure on the gut wall from impacted feces. This is associated with transmural necrosis. We present a 31-year-old pregnant woman who reported abdominal pain and vomiting at 34 weeks of gestation. The diagnosis was unclear from examination and imaging studies, and a provisional diagnosis of acute appendicitis was made. The patient underwent laparotomy and was found to have fecal contamination and multiple stercoral ileal perforations. The bowel segment was resected and exteriorized as a stoma.

## Introduction

Stercoral perforations in the gut are usually a rare cause of inflammatory ailments, which augment pressure-induced transmural necrosis due to a fecaloma or impacted fecalith. Stercoral colitis, the most common form of this spectrum, has a mortality rate of 32-57% [1]. In developing countries like India, tubercular and typhoid perforations in the ileum are common and present with generalized peritonitis. Terminal ileum perforation presenting as obscure peritonitis is rare. They are foreshadowed by an exacerbation of abdominal pain in the right iliac fossa, voluntary guarding, and rigidity. Contrast-enhanced computed tomography (CT) of the abdomen is an excellent modality to pick up extraluminal free air. However, without extraluminal free air, imaging studies may or may not show a clear diagnosis and should be continuously aided by clinical findings and diagnosis [2]. Especially for pregnant women, the diagnosis becomes more difficult. Such patients can undergo a diagnostic laparoscopy for a definitive diagnosis and proceed accordingly. We present a 31-year-old pregnant woman with stercoral ileal perforation who was initially suspected to have acute appendicitis, and a possible pathological mechanism for forming stercoral ileal perforation is hypothesized.

## Case presentation

A 31-year-old pregnant woman, 34 weeks of gestation, complained of vomiting for the last two days and obstipation for the last five days. A total of six to seven episodes of vomiting per day containing 250 mL of non-blood-stained fluid were experienced. She also complained of abdominal pain for three days, which was diffuse and more in the right lower quadrant of the abdomen. There was no history of medical comorbidities. On examination, she was mildly pale, dehydrated, and afebrile. Her vitals were stable, with a pulse of 82 beats per minute and a blood pressure of 110/70 mmHg. An abdominal examination revealed tenderness in the right iliac fossa with severe guarding and localized rigidity. All other systemic examinations were within normal limits. A rectal examination was normal. Obstetric examination revealed a single fetus in cephalic presentation with a fundal height of 34 weeks and a high floating head.

**Figure 1:**
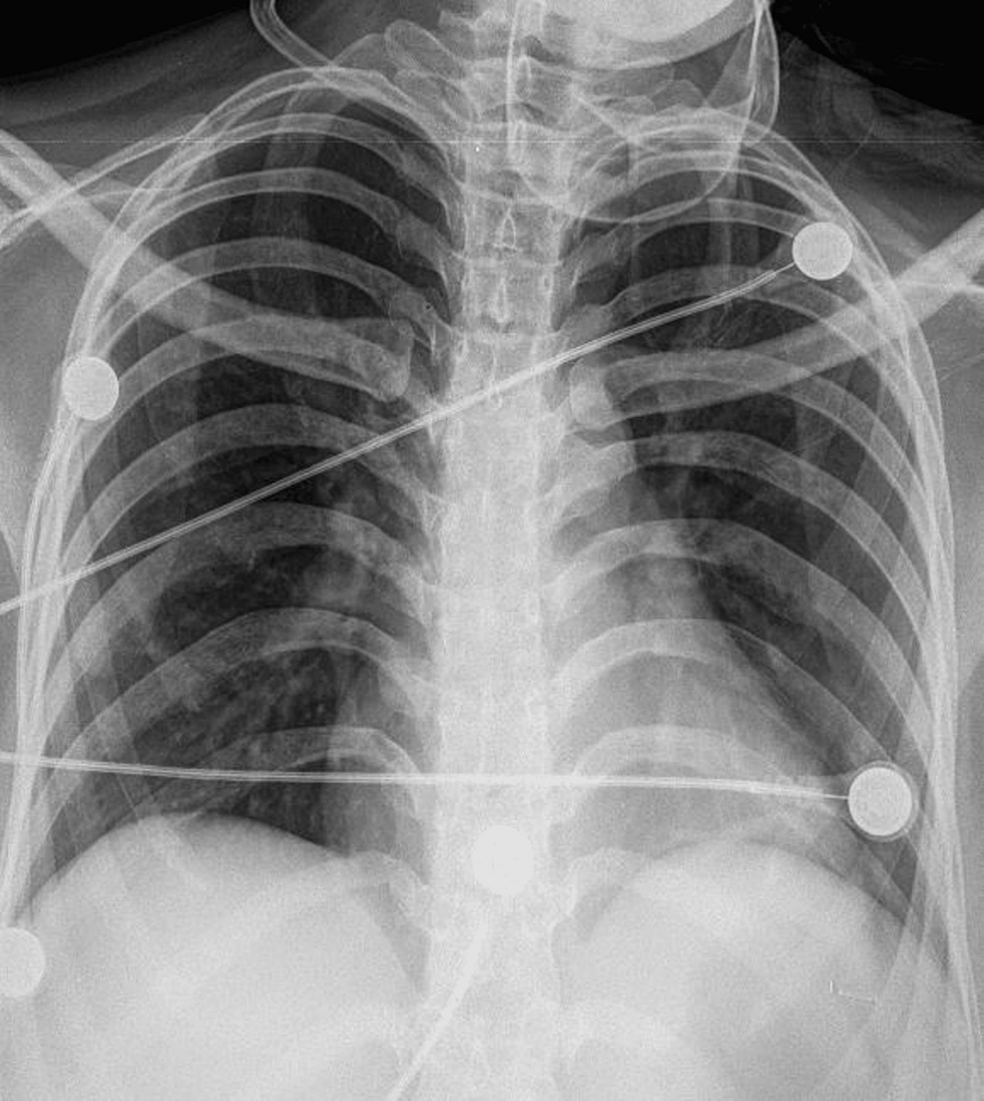
Chest X-ray does not show air under the diaphragm.

Blood investigations showed mild anemia with a hemoglobin of 9.8 g/dL and normal white blood cell counts. Liver function tests and renal function tests were normal. A chest X-ray showed no air under the diaphragm (Figure 1).

**Figure 2:**
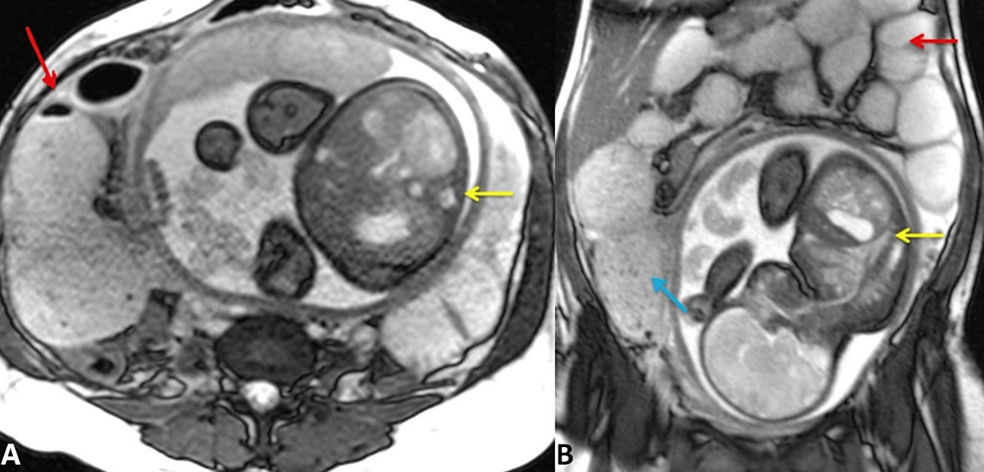
MRI showing (A, axial view) a single intrauterine fetus (yellow arrow) and a dilated segment of the ileum in the right iliac fossa (red arrow) and (B, sagittal view) a dilated segment of the ileum in the right iliac fossa (blue arrow), a single intrauterine fetus (yellow arrow), and a dilated small bowel (red arrow). MRI: magnetic resonance imaging.

Ultrasonography (USG) of the abdomen showed dilated small bowel loops. There was no evidence of collection, growth, or mesenteric lymphadenopathy. The appendix was not visualized. Magnetic resonance imaging (MRI) showed gross dilatations of the small bowel loops with a maximum caliber of 6 cm in the ileum and a mildly distended cecum. Fat stranding around the ileal loop with minimal free fluid in the abdomen was present. There was no evidence of extraluminal free air. Impacted stool in the ileum could not be clearly commented upon when interrogated retrospectively in the postoperative period. There was a single intrauterine fetus. The appendix could not be visualized (Figure 2).

With clinical examination and imaging findings, we had three probable differential diagnoses: perforated appendicitis, perforation peritonitis, and intestinal obstruction.

**Figure 3:**
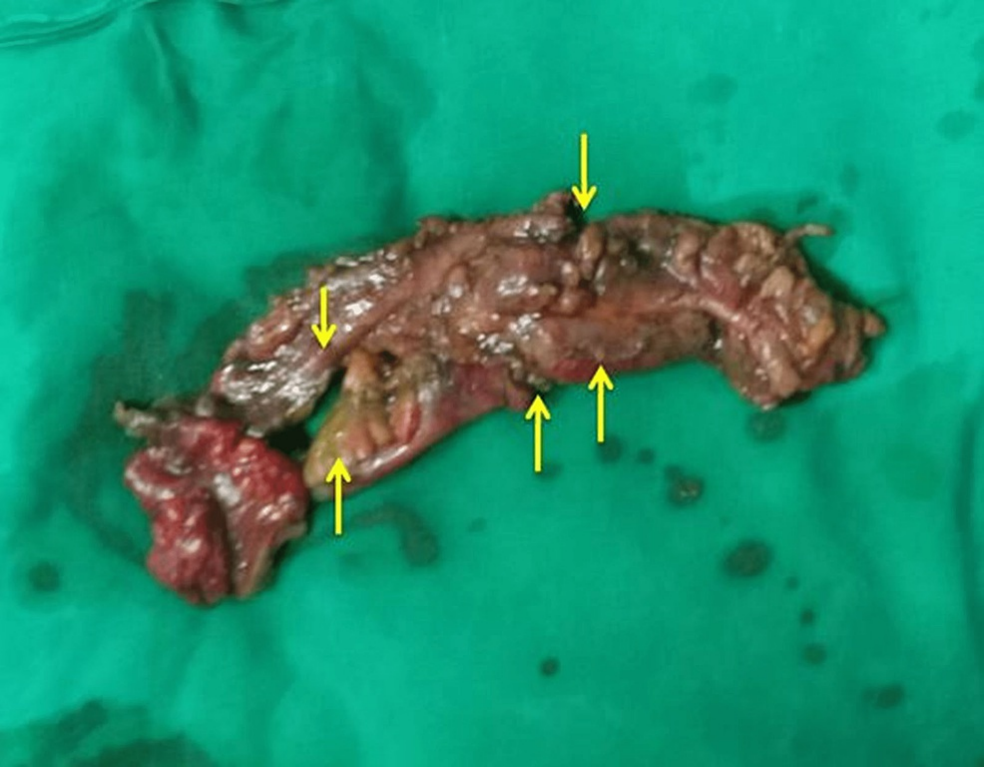
A resected segment of the ileum with multiple perforations in the wall (arrows).

Considering these differential diagnoses in mind and acute appendicitis being the most common surgical emergency in pregnant patients, we planned the patient for an emergency laparotomy. There was fecal contamination of the abdominal cavity in the right iliac fossa, around 150-200 mL. Rest of the peritoneal cavity, fecal contamination was not present. Multiple perforations, measuring 0.5 cm x 0.5 cm, are noted nearly 40 cm from the ileocecal junction (Figure 3).

The appendix was normal and in a retrocecal position. The segment of the ileum with perforation had hard and impacted stools. Hence, the bowel with multiple perforations was resected, about 30 cm long, and an ileostomy was done. After the resection of the segment of the ileum with multiple perforations, the remaining distal ileum was brought as a distal mucus fistula. Fetal delivery was performed by lower segment cesarean section (LSCS).

Post-operative biopsy of the resected specimen showed transmural necrosis and attenuation of the ileal wall, along with an acute inflammatory infiltrate covered with neutrophilic exudate and inflammatory granulation tissue. The inflammatory infiltrate is transmural and reaches the serosal surface. There is no evidence of granulomas, organisms, or parasites in the studied sections. There was no evidence of vessel thrombosis. She was evaluated with the Widal test and cartridge-based nucleic acid amplification test (CBNAAT), which were negative. She was followed up for around one month with no post-surgical complications. 

## Discussion

Ileal perforations are a common surgical emergency in developing countries. Infectious etiologies have been found to be predominant, with typhoid being the most common, followed by tuberculosis, trauma, and nonspecific enteritis [2]. According to the most available literature regarding ileal perforations, the most affected age group is 35-40 years. Increasing mortality and morbidity may be due to poor immune defense and comorbid conditions. Additionally, it is more prevalent among males, contributing to around 75% of all reported cases [3]. Our patient was a young pregnant woman without comorbidity or immunocompromisation.

Stercoral ulceration is the loss of bowel integrity from inspissated feces pressure effects. The risk factors for stercoral perforations include chronic constipation, male sex, older age, nursing facility residence, use of constipating medications, and evidence of fecal impaction [4]. Fecal impaction induces centrifugal pressure, thereby initiating inflammation in the bowel wall. This is followed by ischemia and transmural necrosis of the bowel wall. This phenomenon is predominantly precipitated by stationary accumulated stool in the gut for a prolonged period. This causes the stool to harden due to critical time-dependent water resorption, impairing mass-based contact pressure [5].

Most stercoral perforations are seen in the sigmoid and rectosigmoid, occurring in 77% of cases [6]. This is supported by the fact that the rectosigmoid has the smallest luminal diameter, exerts the highest intraluminal pressure, and has the least amount of water. Rarely, the dilated bowel compromises blood flow to the colon and may present with ischemic colitis [7]. Contrary to the established notion, our patient presented with multiple non-infective and non-traumatic stercoral ileal perforations. We hypothesize that the gravid uterus compressing the small bowel caused the formation of a pseudo-closed loop syndrome with already existing fecal content inside. This content has been dehydrated enough over time to confer the same pathomechanism as stercoral colitis, leading to multiple inflammatory ileal perforations.

Perforations typically manifest with abdominal pain, which localizes to the nearest domain of the perforation. They also present with guarding and rigidity, with or without rebound tenderness. Depending on the amount of fecal contamination, a patient with a small bowel perforation can present with completely stable hemodynamics. On the contrary, few patients present with severe septic shock and require ventilator support. Patients with stercoral ulcers may present either way. Our patient presented with abdominal pain and vomiting but with stable vitals. However, pregnancy was a special challenge for us to manage and prevent fetal or maternal mortality.

Most patients with ileal perforation peritonitis have just one or two perforations, whereas the presence of patients with multiple perforations suggests the possibility of an immunocompromised state [8]. Uncommon causes of ileal perforation include dengue, acute diverticulosis, and prolonged steroid therapy [9,10]. When multiple, the perforations range from 0.5 to 1.5 cm in most cases on the antimesenteric border. They are located mainly within 5-60 cm of the ileocecal junction [11]. There has been a significant increase in mortality of 32-57% for patients with delayed presentation of more than two days, multiple perforations, and fecal peritonitis [1]. Our patient had multiple ileal perforations with impacted stools in the bowel loop.

The diagnosis of ileal perforation might be difficult in atypical situations. A simple erect chest X-ray demonstrates the air under the diaphragm, which reveals perforation. However, the perforation site cannot be determined. Air under the diaphragm may also be absent due to a sealed-off perforation. In stercoral ileitis, the perforation might have been sealed off, which made the presentation atypical and therefore required higher investigations for a proper diagnosis. Contrast-enhanced CT of the abdomen is required for diagnosis. CT shows a hyperemic bowel wall, ascites, and extraluminal air in the peritoneal cavity. The absence of intraperitoneal fluid does not rule out bowel perforation [12]. An MRI of the abdomen should be done in patients where CT is contraindicated or if the patient is pregnant. In our patient, we did an MRI abdomen, as there was no evidence of air under the diaphragm on the chest X-ray.

The extensivity of the intrabdominal fecal contamination, the general condition of the patient, and the location of the ileal perforation are the determining factors for the type of surgical intervention. A diversion ileostomy is an effective option, as patients usually present with abdominal contamination. It is the procedure of choice in cases with delayed presentation (more than one day) after perforation, hemodynamic instability, extensive contamination with severe enteritis, multiple perforations, and signs of impending perforation.

Suppose the involved bowel segment shows signs of impending or multiple perforations within a segment. In that case, segment resection is warranted. Following this, the fate of the resected ends is based on the overall clinical scenario. As mentioned above, these ends can be anastomosed or exteriorized as a stoma. In our case, the patient had delayed presentation and multiple perforations. The involved segment was resected with an ileostomy. To our knowledge, stercoral ileal perforation peritonitis has never been reported in the medical literature. Hence, this would help clinicians have a high suspicion of an early entity diagnosis.

## Conclusions

Stercoral ileal perforations are rare and have never been documented in the literature. This is the first case reported in pregnant women. The clinical picture may mimic acute appendicitis. Imaging studies help with diagnosis; however, sometimes, the findings are more obvious, and diagnosis may be delayed. Such patients will be ideal candidates for diagnostic laparoscopy and will be treated accordingly. Surgical treatment is the only way to reduce mortality, including segmental bowel resection and ileostomy.
